# An Update on AMPK in Hydrogen Sulfide Pharmacology

**DOI:** 10.3389/fphar.2017.00810

**Published:** 2017-11-08

**Authors:** Minjun Wang, Wenbo Tang, Yi Zhun Zhu

**Affiliations:** ^1^Department of Pharmacology, School of Pharmacy, Macau University of Science and Technology, Macau, China; ^2^Shanghai Key Laboratory of Bioactive Small Molecules, Department of Pharmacology, School of Pharmacy, Fudan University, Shanghai, China; ^3^Department of Oncology, School of Medicine, Fudan University, Shanghai, China; ^4^Department of Medical Oncology, Fudan University Shanghai Cancer Center, Shanghai, China

**Keywords:** hydrogen sulfide, AMPK, pharmacology, cardiovascular, autophagy, inflammation, oxidative stress

## Abstract

Hydrogen sulfide (H_2_S), the third bio-active gasotransmitter, is produced endogenously and tightly involved in the pathogenesis and treatment for various diseases. Adenosine 5′-monophosphate-activated protein kinase (AMPK) plays a paramount role in maintaining cellular energetic balance. Increasing evidences have also suggested AMPK as a novel modulator in multiple pathological conditions. In this paper, we will review the biological principles of H_2_S and AMPK, and most importantly, the recent discoveries regarding AMPK-mediated pharmacological actions of H_2_S. Emphasis will be laid on AMPK/H_2_S interactions in the cardiovascular system, autophagy, diabetic complications, and inflammation. In most cases described in this article, by promoting AMPK activation, H_2_S exerts cytoprotective effects or therapeutic potentials, though there remain some controversies before we can fully understand the involved mechanisms. Further researches are in need to investigate more closely any relationship between H_2_S and AMPK, and to put forward the development of H_2_S donors for clinical application.

## Introduction

Hydrogen sulfide (H_2_S), the gas with “rotten egg” smell, has been recognized as the third bio-active gasotransmitter after nitric oxide and carbon monoxide. Mounting studies have revealed its protective effects in the cardiovascular system, central nervous system as well as in diabetes and inflammation. Besides the prominent role in metabolic regulation, recent reports have suggested that adenosine 5′-monophosphate (AMP)-activated protein kinase (AMPK) also participates in various physiological and pathological processes and functions as a critical mediator in the effects of H_2_S. In this paper, we will review the latest and emerging evidences on AMPK-mediated therapeutic potentials of H_2_S.

## Principles of H_2_S Biology

### Biosynthesis of H_2_S

For 100s of years H_2_S was thought to be noxious and toxic. What’s interesting is that H_2_S and organosulfur compounds could be easily found in recipes, such as garlic products. Dietary garlic is well-known for its benefits in lowering blood pressure and lipid levels (Jung et al., 2014; Ried, 2016). In fact, Benavides et al. (2007) pointed out that garlic-derived organic polysulfides were converted rapidly by red blood cell into H_2_S, which was responsible for the subsequent vasorelaxation effects.

In addition to dietary consumption, H_2_S is endogenously produced as well. Physiological H_2_S production in mammal cells is mainly attributed to three enzymes, including cystathionine β-synthase (CBS), cystathionine γ-lyase (CSE), and 3-Mercaptopyruvate sulfurtransferase (3MST).

All these enzymes exhibit tissue-specific expression. CBS is expressed abundantly in the central nerves system, liver, kidney, and so on (Fiorucci et al., 2006; Kimura, 2013; Feliers et al., 2016). It was believed that CSE was mainly produced in the cardiovascular system (Polhemus and Lefer, 2014), but recent studies reported that CSE was also detected in the liver, lung, and kidney (Song et al., 2014; Wang et al., 2017). 3MST was originally discovered in the brain of *Cbs* knockout mice (Shibuya et al., 2009), and further confirmed as a ubiquitous enzyme expressed in the lung, kidney, liver, and vasculature (Ahmad et al., 2016).

Moreover, these enzymes differ in cellular localization and H_2_S metabolism. CBS and CSE are localized in the cytosol, while 3MST is produced in both the cytosol and mitochondrial (Kimura, 2011). In contrary to 3MST, CBS, and CSE are involved in multiple transsulfuration reactions, with pyridoxal 5′-phosphate as a cofactor and sulfur amino acids as substrates, including L-cysteine, L-cystine, and homocysteine (Li et al., 2011). The H_2_S metabolism has been reviewed in detail elsewhere by Wang (2012).

### Pharmacological Actions of H_2_S

During the past decades, increasing studies have evidenced the functions of H_2_S in mammals, among which cardiovascular regulation is the most investigated (Polhemus and Lefer, 2014). *Cse* knockout leads to severe hypertension in mice, suggesting H_2_S as a potential vasodilator (Yang et al., 2008). Indeed, similar to the other two gasotransmitters, both endogenous and exogenous-applied H_2_S exhibits vasorelaxation effects in multiple types of blood vessels (Kiss et al., 2008). It was reported that the proliferation of vascular smooth muscle cell was inhibited by H_2_S, as opposed to vascular endothelial cells (Du et al., 2004; Papapetropoulos et al., 2009). Furthermore, H_2_S protects mice from myocardial infarction and ischemia-induced heart failure (Calvert et al., 2010; Miao et al., 2016).

Apart from the cardiovascular system, H_2_S also contributes to protective effects in inflammation, oxidative stress, and nervous system. Despite the controversy regarding the multiple roles of H_2_S in inflammatory disorders, it is generally accepted that H_2_S ameliorates neuroinflammation, suppresses inflammatory cytokine production, and inhibits activation of key transcriptional factors. More details about the physiological and pharmacological functions of H_2_S could be found in reviews by Li et al. (2011) and Wang (2012).

## AMPK and its Biological Function

### AMPK Regulation

Adenosine 5′-monophosphate-activated protein kinase is a conserved energetic sensor existed in almost all eukaryotes. Composed of a catalytic α-subunit and regulatory β- and γ-subunits, AMPK monitors intracellular AMP and adenosine triphosphate (ATP) levels (Kahn et al., 2005; Li et al., 2015). In mammals, APMKα catalytic subunits and β-subunits are encoded by two genes separately (α1, α2, and β1, β2), and γ-subunit by three genes (γ1, γ2, and γ3), making at least 12 possible heterotrimer combinations, of which the expression may be tissue restricted (Hardie et al., 2012). For example, γ3 isoform is dominantly expressed in skeletal muscle, and both the wild type and a mutation of arginine to glutamine at position 225 (R225Q) in the CBS domain results in increased glycogen concentrations in skeletal muscle of the transgenic mice (Yu et al., 2006).

During energy deprivation characterized by increased AMP/ATP ratio, AMPK is phosphorylated at Thr172 and allosteric activated with a 100-fold increase in kinase activity (Hawley et al., 1996). In mammals, the typical kinases involved in canonical AMPK activation include liver kinase B1 (LKB1) and Ca^2+^/calmodulin-activated protein kinase kinase β (CaMKKβ). LKB1, also known as a potent tumor suppressor, is vital for the basal phosphorylation level of AMPK (Hardie et al., 2012). Genetic knockout of *LKB1* impairs AMPK activation by AMP, indicating its critical role during energy deprivation (Hardie and Alessi, 2013). AMPK is also activated in response to calcium flux, which relies on the intact function of CaMKKβ. It is noteworthy that this alternative AMPK activation pathway is independent of any change in cellular AMP level (Hardie et al., 2012), and is most highlighted in the brain (Green et al., 2011).

### AMPK Activators

In addition to cellular metabolic signal, AMPK can be activated by a variety of compounds. AICAR (5-amino-4-imidazolecarboxamide riboside-1-β-D-ribofuranoside) is a potent AMPK activator. As an adenosine analog, AICAR is taken in by adenosine transporters and converted into ZMP (5-aminoimidazole-4-carboxamide-1-β-D-furanosyl 5′-monophosphate). Similar to AMP, ZMP binds directly to AMPK and induces its allosteric activation (Wong et al., 2009). Natural products and related derivatives represent another class of AMPK activators. Metformin, derived from French lilac, is widely administrated as the first-line medication for type 2 diabetes. Although the involved mechanisms remain partially understood, it is clear that AMPK plays an important role in the benefits of metformin (Zhou et al., 2001). Resveratrol, isolated from grapes and red wine, is reported to activator AMPK and SIRT1, a NAD^+^-dependent protein deacetylase sharing crosstalk with AMPK (Price et al., 2012). Other natural AMPK activators include epigallocatechin gallate, capsaicin, curcumin, berberine, and so on (Hwang et al., 2005; Ejaz et al., 2009; Jeong et al., 2009).

### AMPK on Cellular Metabolism and More

As a master cellular energy gauge, AMPK regulates lipid and glucose metabolism by phosphorylating downstream targets. Activated AMPK not only suppresses fatty acid synthesis by impairing acetyl-CoA carboxylase (ACC), but also facilitates lipid oxidation by boosting malonyl-CoA decarboxylase (MCD) (Zang et al., 2004; Zhang et al., 2009). Similar regulating pattern is also observed in glucose metabolism. By promoting the translocation of glucose transporter 4 (GLUT4), AMPK activation stimulates glucose uptake in muscle tissue (Jäger et al., 2007). In parallel, hepatic gluconeogenesis is inhibited by AMPK through decreased phosphoenolpyruvate carboxykinase (PEPCK) and glucose-6-phosphatase (G6Pase) transcription (Zhang et al., 2009). In general, AMPK activation leads to metabolic changes toward relieving energy deprivation.

In addition to energy balance, recent studies have suggested the participation of AMPK in autophagy, atherosclerosis, inflammation, and cancer, which shares crosstalk with H_2_S (Motoshima et al., 2006; Kim et al., 2011; O’neill and Hardie, 2013). The advances in AMPK researches have shed light on novel therapeutic potentials of AMPK activators and H_2_S donors, and provided modern understanding of metabolism. More details will be discussed in the following sections.

## AMPK in H_2_S Pharmacology

Great efforts have been made to elucidate the roles of AMPK in H_2_S pharmacology, in which AMPK usually serves as a key mediator. Despite the fact that AMPK was reported to be inhibited by H_2_S in some circumstances (Zhang et al., 2012), it is generally accepted that H_2_S exerts its biological activity by activating AMPK. In this part we will review the recent discoveries on AMPK-H_2_S pharmacology.

### AMPK in Cardioprotective Effects of H_2_S

According to latest Global Burden of Disease Study, ischemic heart disease remained as the global leading cause for death in 2010, accounting for 13.3% of total death worldwide (Lozano et al., 2013). A great number of patients are suffering from related diseases including ischemic heart failure and stable arrhythmia (McMurray et al., 2005). The beneficial effects of H_2_S on survival after cardiac arrest in mice was first reported in 2009. By *i.v.* application of Na_2_S, an exogenous H_2_S donor, 7 min following cardiac arrest, significant increase in survival rate was achieved compared with vehicle (Minamishima et al., 2009). The authors pointed out that the treatment effects of Na_2_S was associated with activated AMPK prosurvival signals. In addition, AMPK is also attributed to the cardioprotective effects of H_2_S in high fat diet-induced cardiac dysfunction and impaired left ventricular function by smoking (Zhou et al., 2014a; Barr et al., 2015).

Myocardial ischemia/reperfusion (I/R) injury is a complication of inflammation and oxidative damage encountered when restoring blood supply of ischemic regions. Previous studies have demonstrated that H_2_S protects from I/R injury by multiple mechanisms, including ameliorating oxidative stress, inflammation, and apoptosis (Sivarajah et al., 2009; Meng et al., 2015). Xie et al. (2015) reported that AMPK is a critical mediator in the effects of H_2_S donor ADT. By maintaining autophagy flux impaired by I/R, as evidenced by reduced LC3-II/LC3-I ratio and beclin-1 expression, H_2_S-activated AMPK significantly relieved myocardial I/R injury.

Post-conditioning (PC), defined as brief repeated periods of ischemia performed at the onset of reperfusion, has been proved to reduce I/R injury in both cardiomyocytes and coronary vascular endothelium cells (Vinten-Johansen et al., 2005). Nevertheless PC only exerts cardioprotection in young but not old hearts (Boengler et al., 2009). Chen et al. (2016) suggested that by exogenous application of H_2_S, the treatment effects of PC could be restored in isolated aged rat hearts and aged cardiomyocytes. In contrast to the study on I/R, stimulated AMPK promoted autophagy, which subsequently decreased apoptosis, reduced myocardial injury, and improved cardiac function. More studies regarding AMPK-H_2_S and autophagy will be discussed in the next section.

### AMPK-Regulated Autophagy in H_2_S Pharmacology

Autophagy is a lysosome-dependent “self-engulfment” process in which cells digest their own cytosolic components to maintain metabolic homeostasis during starvation. Moreover, autophagy is tightly involved in cancer, cardiac and liver diseases (Levine and Kroemer, 2008). The autophagy pathway was originally discovered with Atg1/UNC-51-like kinase (ULK) 1 complex as an essential initiator, which also senses cellular nutrient status from the mammalian target of rapamycin (mTOR). Previous studies have established the prominent role of AMPK in regulating autophagy. One of the most described mechanism involves the suppression of mTOR pathway by AMPK, which promotes the formation of autophagosomes (Kim and Guan, 2015). On the other hand, AMPK directly phosphorylates several critical sites in ULK1 and subsequently induces its activation in autophagy and mitochondrial homeostasis (Hardie, 2011). By inhibiting mTOR and activating ULK1, AMPK regulates autophagy in response to nutritional signal (Mihaylova and Shaw, 2011).

The AMPK-autophagy pathway has been identified in multiple pharmacological functions of H_2_S. As described in the last section, H_2_S mitigates cardiac I/R injury and restores PC protection by regulating AMPK-mediated autophagy. Similar results were also observed in high glucose conditions. Vascular endothelial dysfunction induced by hyperglycemia impairs vasodilation and angiogenic function, leading to diabetic complications (Hink et al., 2001). Exogenous H_2_S markedly preserved arterial endothelial cells by reducing AMPK phosphorylation and inhibiting excessive autophagy (Liu et al., 2016). Furthermore, Kundu et al. (2014) suggested that H_2_S relieved renal matrix accumulation during hyperglycemia by LKB1/AMPK cascade. Intriguingly, as opposed to the previous study, AMPK was activated by H_2_S, leading to protective autophagy in glomerular endothelial cells (Kundu et al., 2014).

Hypertriglyceridemia is among the most common metabolic diseases and is proved as an independent risk factor for cardiovascular and cerebrovascular events (Chapman et al., 2011). Lower plasma H_2_S content was reported to be associated with dyslipidemia, indicating the potential regulation of H_2_S on serum triglyceride (Liu et al., 2006). Indeed, Sun et al. (2015) claimed that H_2_S reduced triglyceride and relieved non-alcoholic fatty liver disease in mice by activating AMPK and inhibiting mTOR. Consistent regulation of AMPK and autophagy by H_2_S was reported in inhibiting colon epithelial cell proliferation (Wu et al., 2012).

### AMPK in H_2_S Protection against Diabetic Complications

Chronic hyperglycemia of diabetes leads to damage and dysfunction of multiple organs, including the kidney, heart, and blood vessels (American Diabetes Association, 2014). The anti-diabetic effects of AMPK activators are well-documented, and numerous evidences have indicated the mitigation of hyperglycemia and related complications by H_2_S (Suzuki et al., 2011; Szabo, 2012). Lee et al. (2012, 2017) suggested that H_2_S decreased protein synthesis, cellular hypertrophy, and matrix protein accumulation of renal cells under high glucose condition. Moreover, H_2_S inhibited mTOR activity, mRNA initiation and elongation by activating AMPK through CaMKKβ. Corresponding AMPK/mTOR regulation by H_2_S was also reported in cardiomyocyte protection from high glucose (Wei et al., 2014).

Vascular inflammation induced by hyperglycemia is associated with impaired insulin sensitivity and accelerated atherosclerosis (Basta et al., 2004; Paneni et al., 2013). Both L-cysteine, the endogenous precursor of H_2_S, and exogenous H_2_S donors successfully diminished high glucose-stimulated inflammatory cytokine secretion from monocytic cells, indicating the link between H_2_S and vascular inflammation (Jain et al., 2010). It was further supported that H_2_S relieved vascular inflammation via multiple mechanisms including activating AMPK (Manna and Jain, 2013). These findings might bring light on the therapeutic potentials of H_2_S against diabetic complications.

### AMPK in Anti-inflammatory and Anti-oxidant Stress Properties of H_2_S

There have been controversies over the exact role of H_2_S in inflammation (Whiteman and Winyard, 2011). Despite distinct results obtained, it appears that the inconsistency in the pro- and anti-inflammatory effects of H_2_S might be attributed to models, doses, and sampling time (Hegde and Bhatia, 2011). It is generally acknowledged that H_2_S shows remarkable suppression on lipopolysaccharide-induced production of inflammatory cytokines both in macrophages and microglia cells (Hu et al., 2007; Whiteman et al., 2010). In parallel, the inhibition of inflammation by AMPK activation has been also been reported (O’neill and Hardie, 2013). Zhou et al. (2014b) found that the suppression of microglia inflammation by H_2_S was largely dependent on AMPK activation via CaMKKβ pathway, which was evidenced by multiple H_2_S donors, CBS overexpression, and *AMPK* knockdown.

Anemia of inflammation (AI) is the second prevalent anemia and a common complication in patients with chronic diseases (Weiss and Goodnough, 2005). Lower hemoglobin is associated with increased mortality in diseases such as heart failure (Horwich et al., 2002; O’Meara et al., 2006), cancer (Caro et al., 2001), and chronic kidney disease (Foley et al., 1996; Vlagopoulos et al., 2005). The pathological changes of AI include iron disturbance and erythroid system dysfunction (Nemeth and Ganz, 2014), of which iron dysregulation is the hallmark. Mounting evidences have indicated that elevated hepatic and circulating hepcidin, a liver-derived iron-regulating peptide, is tightly involved in the progression of AI (Ganz, 2003), making it an ideal target for AI treatment. We first reported that during inflammation H_2_S inhibited hepatic hepcidin, a critical factor in AI pathogenesis (Xin et al., 2016). Our recent investigation revealed that the effects of H_2_S was partially mediated by AMPK (Wang et al., 2017). What’s more, both pharmacological and genetic activation of AMPK, as well as metformin, ameliorated chronic AI in mice, which was supported by our clinical samples and independent groups (Kim et al., 2016; Wang et al., 2017). Interestingly, metformin was reported to increase H_2_S content in various mouse tissues (Wiliński et al., 2013). Much more work will be needed to broaden our understanding about the interaction among H_2_S, AMPK and metformin.

5′-Monophosphate-activated protein kinase also contributes to the effects of H_2_S and garlic products against oxidant stress. Han et al. (2011) claimed that ajoene, a garlic by-product, reduced oxidative injury and hepatic steatosis by stimulating LKB1/AMPK pathway. Consistent results were observed in H_2_S donors. Sodium hydrosulfide, an inorganic H_2_S donor, ameliorated oxidative stress and apoptosis via activating CaMKKβ/AMPK signaling, finally attenuating experimental aging process (Chen et al., 2017). Furthermore, reactive oxygen species production was diminished by H_2_S-induced AMPK activation in osteoblast cells treated with dexamethasone (Yang et al., 2014).

## Current Research Gaps

It is of great importance to understand the specific mechanisms within H_2_S-induced AMPK activation. However, few studies have looked into this topic. Kundu colleagues claimed that H_2_S activated AMPK by LKB1 (see section “AMPK in Cardioprotective Effects of H_2_S”), while Lee colleagues (see section “AMPK-Regulated Autophagy in H_2_S Pharmacology”) and Zhou colleagues (see section “AMPK in H_2_S Protection against Diabetic Complications”) suggested CaMKKβ was indispensable for AMPK activation by H_2_S.

It is worth noting that instead of fully elucidating the potential mechanisms, all these studies mainly pointed out several key mediators in the H_2_S-AMPK pathway. Moreover, recent studies have revealed that H_2_S might also regulate protein functions in a more “direct” manner, such as by sulfhydration (Paul and Snyder, 2012) and forming polysulfides (Greiner et al., 2013; Kimura et al., 2013). Future studies may help better understand the role of these novel modifications in H_2_S-AMPK interactions.

## Conclusion

**FIGURE 1:**
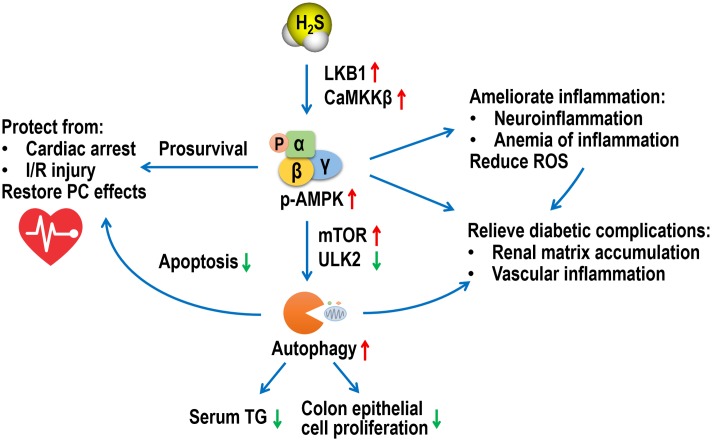
A schematic diagram of multiple roles of AMPK in the pharmacological functions of H_2_S. AMPK, adenosine 5′-monophosphate-activated protein kinase; H_2_S, hydrogen sulfide; LKB1, liver kinase B1; CaMKKβ, Ca^2+^/calmodulin-activated protein kinase kinase β; mTOR, mammalian target of rapamycin; ULK2, UNC-51-like kinase 2; TG, triglyceride; I/R, ischemia/reperfusion; PC, post-conditioning; ROS, reactive oxygen species.

In summary, this minireview provides novel insights into latest AMPK-mediated H_2_S pharmacology in various tissues and diseases (**Figure 1**). Despite substantial progress, there remains a long road toward complete understanding of AMPK-H_2_S interactions and application of H_2_S donors in clinical settings.

## Author Contributions

All authors listed have made substantial, direct and intellectual contributions to the work, and approved it for publication.

## Conflict of Interest Statement

The authors declare that the research was conducted in the absence of any commercial or financial relationships that could be construed as a potential conflict of interest.
